# Maternal Behaviors during Pregnancy Impact Offspring Obesity Risk

**DOI:** 10.1155/2011/985139

**Published:** 2011-10-26

**Authors:** Suzanne Phelan, Chantelle Hart, Maureen Phipps, Barbara Abrams, Andrew Schaffner, Angelica Adams, Rena Wing

**Affiliations:** ^1^Kinesiology Department, California Polytechnic State University, 1 Grand Avenue, San Luis Obispo, CA 93407-0386, USA; ^2^Department of Psychiatry and Weight Control and Diabetes Research Center, Warren Alpert Medical School of Brown University and The Miriam Hospital, 197 Richmond Street, Providence, RI 02906, USA; ^3^Department of Obstetrics and Gynecology, Warren Alpert Medical School of Brown University and Women and Infants Hospital of Rhode Island, 101 Dudley Street, Providence, RI 02905, USA; ^4^School of Public Health, University of California, Berkeley, 140 Warren Hall, Berkeley, CA 94720, USA; ^5^Statistics Department, California Polytechnic State University, 1 Grand Avenue, San Luis Obispo, CA 93407, USA

## Abstract

This study investigated the effects of maternal changes during pregnancy in diet, exercise, and psychosocial factors on offspring weight parameters at birth and 6 months. In overweight/obese (OW/OB; *n* = 132) mothers, greater % kcal from sweets early in pregnancy was the strongest, independent predictor of higher weight for age (WFA) (beta = 0.19; *P* = 0.004), higher odds of macrosomia (OR = 1.1 (1.0–1.2); *P* = 0.004) andWFA *>*90th percentile at birth (OR = 1.2 (1.1–1.3); *P* = 0.002) and higher WFA at 6 months (beta = 0.30; *P* = 0.002). In normal weight (*n* = 153) mothers, higher intake of soft drinks was the strongest predictor of higher offspring WFA at birth (beta = 0.16; *P* = 0.04) but not at 6 months. Prenatal physical activity, depressive symptoms, and sleep-related variables did not significantly predict offspring weight outcomes. Mothers' eating behaviors during pregnancy, especially intake of sweets in OW/OB mothers, may have a lasting effect on child weight.

## 1. Introduction

Risk of obesity is shaped, in large part, by early life events starting during gestation. One powerful early life exposure is maternal obesity during pregnancy. Studies have consistently found that higher maternal weight entering and during pregnancy increases risk for obesity among offspring in childhood, adolescence, and adulthood [1, 2]. While shared genes account for some of the similarity in maternal and offspring weight, evidence suggests that exposure to the uterine environment of an obese woman itself may directly program offspring obesity [3].

Another independent determinant of offspring weight status is gestational weight gain (GWG). Both excessive [4] and inadequate [5] weight gain in the mother during gestation have been shown to lead to overly rapid fetal and infant growth rates and the programming of future risk of childhood overweight and metabolic disease. In the USA excessive GWG remains of predominant concern, as 60% of obese women gain more than recommended. Also, approximately 40% of normal weight women gain more than recommended [6], increasing the risk of obesity in their offspring. 

Although maternal obesity and excessive GWG are both the result of energy intake exceeding energy expenditure, few studies have examined the specific influences of maternal dietary and exercise behaviors during pregnancy on offspring obesity risk. Some evidence suggests that maternal prenatal intake of protein and fat [7–9] may be positively associated with birth weight. In animal models, prenatal exposures to overnutrition, high-fat and high-protein diets, “junk” food, and stress have been linked with greater adiposity in offspring [10–12]. In nonpregnant humans, intake of soft drinks and junk food [13], higher dietary disinhibition [14], and restrictive dieting practices [15] have been positively correlated with overweight in children. However, little research has comprehensively examined the impact of maternal prenatal behaviors and psychosocial variables measured during pregnancy on subsequent offspring weight status at birth and 6 months. This information is critical for the design of future intervention trials targeting obesity prevention in children. 

The primary aim of this study was to examine the impact of GWG, maternal eating and exercise behaviors, and psychosocial factors on offspring weight status. We hypothesized that offspring exposed to maternal obesity, excessive GWG, poor prenatal eating habits, less exercise in pregnancy, and greater maternal psychosocial distress during gestation would have higher weight for age (WFA) *z*-scores at birth and 6 months than nonexposed counterparts. 

## 2. Materials and Methods

### 2.1. Participants and Procedures

Participants were offspring of women recruited into the Fit for Delivery study, a clinical trial that examined the effects of a lifestyle intervention to reduce excessive GWG in normal weight and overweight/obese women (Clinical Trials no. 01117961) [16]. As reported previously [16], the intervention reduced excessive GWG in normal weight but not overweight/obese women. Normal weight and overweight/obese categories were based on the 1990 IOM cut points [17]: normal weight BMI = 19.8 to 26.0 or overweight/obese BMI 26.1–40.0 [17]. Women were recruited at the time of their first prenatal visit from one of six obstetric offices serving a socioeconomic and ethnically diverse population in Providence, RI from 2006 to 2008. Eligibility criteria included nonsmoking, adults (age >18 years), fluency in English, access to telephone, gestational age between 10 and 16 weeks, singleton pregnancy, no current or history of eating disorders, and without major psychiatric illness (i.e., schizophrenia, bipolar disorder, and panic/anxiety disorder) or major medical problems, including diabetes, stroke, and cancer. Women (*n* = 32) who developed gestational diabetes (GDM) were excluded from analysis a priori, given potential impact of GDM on both offspring and maternal weight gain [18]. All mothers provided written informed consent, and all procedures were conducted in accordance with ethical standards for human experimentation. Mothers were paid $25 for attending the 30-week gestation and 6-month postpartum assessments. The study was approved by the Institutional Review Boards at the Miriam Hospital, in Providence, RI., Women and Infants Hospital of Rhode Island Providence, RI and California Polytechnic State University, in San Luis Obispo, Calif.

### 2.2. Measures

#### 2.2.1. Background Information

At study entry, participants reported via questionnaire information on maternal race/ethnicity, age, education, parity, employment, marital status, and household income. For descriptive purposes, the race variable was defined as non-Hispanic Caucasian, Latina/Hispanic, African-American, and “other.” In other analyses, race was categorized as non-Hispanic Caucasian versus all other groups combined. Income was categorized as >$25,000 per year versus ≤$25,000. Mothers also reported duration of breastfeeding on postpartum questionnaires.

#### 2.2.2. Maternal Anthropometrics

Maternal pregravid weight was based on self-report at time of study enrollment and was shown to be valid compared with physician documented prepregnancy weight [16]. Height was measured by trained research staff using a stadiometer at study entry and was used with pregravid weight to calculate BMI. Total GWG was computed based on pregravid weight and weight at the last clinic visit prior to delivery. Weight at the last clinic visit was objectively measured using a calibrated digital or balance beam scale by research assistant or clinic staff. Data were collected before the 2009 IOM guidelines were released. Based on the 1990 IOM guidelines [17], we classified GWG as “excessive” in normal weight women whose gains were more than 35 pounds (15.9 kg) and overweight (BMI >26 to 29) women whose gains were above 25 pounds (11.4 kg). Similar to other studies, we combined overweight and obese (BMI 29–40) women in our analysis and, thus, also set an upper weight gain goal of 25 pounds (11.4 kg) for these heaviest women [19].

#### 2.2.3. Infant Anthropometrics

Trained research staff abstracted infant and child weight and length from obstetric and pediatric records. We computed birth weight for gestational age *z*-scores using US Natality reference data from Oken et al. [20] and defined small for gestational age (SGA) at birth as WFA *z*-scores <10th percentile and large for gestational age as WFA *z*-scores >90th percentile. Macrosomia was defined as weight >4000 grams [21]. We computed 6-month WFA and sex-specific *z* scores with 2000 CDC reference data [22] and defined risk of obesity as WFA *z*-scores >90th percentile. Other variables abstracted from infant medical records included infant sex and gestational age at birth (calculated from last menstrual period or from second trimester ultrasound if the two estimated differed by >10 days).

#### 2.2.4. Behavioral and Psychosocial Factors during Pregnancy

Self-reported measures of dietary intake and exercise expenditure were obtained by trained assessors at study entry and 30-weeks gestation. The Block Food Frequency questionnaire was used to assess daily caloric intake, calories from sugar-sweetened beverages, and percent intake from fat, protein, carbohydrates, and sweets. This questionnaire has been well validated [23] and validated in pregnancy [24]. Participants completed the questionnaire in reference to intake over the past month.

Fast food consumption was assessed based on self-report questions used in our previous research [25]. Energy expenditure was measured using the Paffenbarger Physical Activity Questionnaire [26], a measure which estimates weekly energy expenditure from self-reports of stairs climbed, blocks walked, and other recreational activities performed in the past week. The Edinburgh Postnatal Depression Scale was used to examine levels of depressive symptoms [27]. The Eating Inventory (EI) [28] was used to assess cognitive restraint and disinhibition. Stress was assessed using the short form of the Perceived Stress Scale [29]. Sleep was assessed using the General Sleep Disturbance questionnaire [30].

### 2.3. Statistics

Both R (version 2.11.1) and SPSS (PASW version 18.0.1) statistical packages were used. Analyses were conducted separately for normal weight and overweight/obese women, given the observed difference in impact of the lifestyle treatment on GWG [16] and effects of maternal prepregnancy weight on offspring weight outcomes [31]. Pearson's product moment correlations were conducted to examine unadjusted associations between WFA *z*-scores and maternal variables. Multiple regression (for continuous outcomes) and logistic regression (for categorical outcomes) were used to examine predictors of infant macrosomia, and WFA *z*-scores at birth and 6 months, and changes in WFA *z*-scores, adjusting for treatment group, infant sex, recruitment clinic, weeks of gestation at delivery, and breastfeeding (in 6-month analyses). Predictors included both baseline and pregnancy changes over time (between entry and 30-weeks gestation). To determine the most robust set of predictors and correlates of infant outcomes, multiple regression analyses were used. To arrive at a final regression model, stepwise analyses were first conducted within predefined categories (i.e., demographic (age, parity, race, marital status, education, prepregnancy BMI), physical activity (calories expended per week in moderate physical activity TV hours per day), macronutrient (% of calories from fat, carbohydrates, protein, sweets, and total calories), dietary components (fast food and sugar-sweetened soft drinks) psychosocial variables (mood, restraint, disinhibition, and stress), and sleep. Variables that were significant or approached significance (*P* < 0.10) within each category in the stepwise analyses were included together in a comprehensive model. Macronutrient (i.e., % kcal from sweets, fat, and protein) and specific dietary components (i.e., fast food or soft drinks) were analyzed in separate models due to their high collinearity. Similarly, as the percentage of calories from fat and carbohydrates was highly correlated (*r* = −.90; *P* = .0001), only the percentage of calories from fat was used in initial modeling. Similar analyses were conducted using logistic regression to examine impact on odds of macrosomia. Prepregnancy BMI was also examined as a categorical predictor (median split within weight groups), but the same results were observed. Exploratory analyses examined whether treatment condition (intervention versus standard care) interacted with dietary variables and physical activity in predicting offspring weight status. After adjustments (infant sex, recruitment clinic, weeks of gestation at delivery, and breastfeeding), no significant interactions were observed in normal weight or overweight/obese mothers (data not shown). 

## 3. Results

### 3.1. Participant Characteristics

Excluding participants with miscarriages (*n* = 6) and GDM (*n* = 32), 363 mothers (177 overweight/obese and 186 normal weight) completed the baseline assessment, which occurred between 10 and 16 weeks of pregnancy (13 weeks on average). At 30 weeks, 341 (94%; 160 overweight/obese and 181 normal weight) attended the assessment visit. Of these, 78.5% (*n* = 285/363; 132 overweight/obese and 153 normal weight) had pediatrician records at birth and 68.6% (*n* = 249/363; 121 overweight/obese and 128 normal weight) had pediatrician information at 6 months. There were no significant differences in retention or availability of pediatrician records in normal weight versus overweight/obese mothers at all time points. Comparing mothers of children with versus without pediatrician records at 6 months, those with pediatrician records were older (29.0 ± 5.1 versus 27.4 ± 5.5 years; *P* = .008), more likely to be non-Hispanic White (73.8% versus 53.0% *P* =  .0001), and more likely to be married (74.6 versus 55.7%; *P* =  .001); no significant differences were observed in prepregnancy BMI, education, income, weeks of gestation at delivery, or maternal GWG.

### 3.2. Maternal BMI and Offspring Weight Parameters

At birth, WFA *z*-scores were significantly higher for offspring of overweight/obese compared with normal weight mothers (0.42 ± 0.92 versus 0.21 ± 0.76, resp.; *P* =  .002). Similarly, a greater proportion of offspring from overweight/obese mothers were classified as macrosomic (>4000 grams; 17.2% versus 4.8%, resp.; *P* =  0.0001) and at >90th percentile at birth (19.6% versus 7.1%; *P* =  .001; Table 1). At 6 months, no significant differences in WFA *z*-scores were observed between offspring of normal weight and overweight/obese mothers. Between birth and 6 months, the percentage of children with WFA >90th percentile declined slightly in overweight/obese mothers (from 19.6% to 17.6%) but increased from 7% to 15% in the offspring of normal weight mothers (Table 1). Subsequent analyses separately examined predictors of offspring weight in normal weight versus overweight/obese mothers. 

### 3.3. Prenatal Predictors of Offspring Weight

#### 3.3.1. Overweight/Obese Mothers

In initial unadjusted analyses, several significant associations between overweight/obese mothers' prenatal behaviors and offspring WFA *z*-scores were observed (Table 2). 

Multiple regression analyses were then conducted to determine the most robust set of predictors of offspring weight parameters after adjustment for treatment group, infant sex, recruitment clinic, weeks of gestation at delivery, and breastfeeding (in 6-month analyses). Examining predictors of birth weight, the strongest predictor of higher WFA z score was consumption of a greater percentage of calories from sweets early in pregnancy (beta = 0.19; *P* =  0.004; Table 3). Similarly, greater consumption of sweets early in pregnancy was significantly related to higher odds of macrosomia (OR = 1.1 (1.0, 1.2); *P* =  0.004) and WFA >90th percentile at birth (OR = 1.2 (1.1, 1.3); *P *=  0.002). Each 1% increase in percentage of calories consumed from sweets early in pregnancy increased the odds of macrosomia by 10% and WFA >90th percentile by 20%.

At 6 months, the strongest predictors of higher WFA *z*-scores were greater percentage of calories from sweets early in pregnancy (beta = 0.30; *t* = 3.2; *P *=  0.002) and also greater increases in percentage of calories from protein during pregnancy beta = 0.20; Table 3). Higher percentage of calories from protein early in pregnancy (OR = 0.38 (0.19, 0.78); *P* =  0.04) was also a significant predictor of reduced odds of WFA >90th percentile at 6 months. 

We examined GWG as an independent predictor and potential mediator of effects of maternal prenatal behaviors on offspring weight outcomes (Table 3). Children of overweight/obese mothers who were exposed to excessive GWG (*n* = 84) had an approximate 4.0-fold increase in odds (CI (1.0, 15.1); *P* =  0.04) of being macrosomic and had higher WFA *z*-scores at birth (0.62 ± 0.84 versus 0.05 ± 0.95; *P* =  0.002) compared with children exposed to adequate GWG. Also, at 6 months, children exposed to excessive GWG had near significant (*P* =  .06) higher odds of WFA *z* score >90th percentile compared with children exposed to adequate weight gain (OR = 6.2 (0.94, 41.1), but no significant association was seen on WFA *z*-scores (0.45 ± 0.14 versus 0.08 ± 0.18; *P* =  .11). When analyzing maternal GWG as a continuous measure, similar results were observed. Total GWG was positively predictive of higher child WFA z score at birth (beta =.41; *t* = 4.9; *P *=  .0001) and macrosomia at birth (OR = 1.2 (1.1, 1.3); *P *=  0.001). At 6 months, no significant effects were seen for GWG on odds of WFA >90th percentile (*P *=  .11) or average *z*-scores (*P *=  .16) at 6 months. 

Examining GWG as a potential mediator, the effects of maternal prenatal behaviors on offspring weight outcomes were generally attenuated (i.e., *P* value increases between 0.01 and 0.06 across analyses) but not entirely removed when GWG was included in the models (Table 3).

### 3.4. Normal Weight Mothers

In initial, unadjusted analyses, few significant correlations were found between prenatal behaviors and WFA *z*-scores at birth and 6 months in the offspring of normal weight mothers. Only lower maternal fat intake (−0.22; *P* = 0.01) and trend for higher carbohydrate intake (0.16; *P* = 0.06) early in pregnancy were correlated with higher WFA *z*-scores at 6 months. 

In final, multiple regression analyses that included adjustments for confounding variables, the strongest predictor of higher WFA z score at birth was greater consumption of soft drinks early in pregnancy (beta = 0.16; *P* = 0.04; Table 4). The omnibus tests of model coefficients predicting macrosomia and WFA >90th percentile at birth were insignificant and few cases of each were observed. 

At 6 months, higher percentage of calories from fat early in pregnancy was a significant predictor of lower WFA *z*-scores (Table 4; beta = −0.35; *P *= 0.001) and reduced odds of WFA >90th percentile (OR = 0.81 (0.70, 0.94); *P* =  .007). Similar, but in the inverse direction, results were observed in models where percentage of calories from carbohydrates (instead of fat) was included (B = 0.04 (0.01, 0.06); beta = 0.27; *P* =.006 for WFA z and OR = 1.1 (1.1, 1.3); *P* = 0.005 for WFA >90th percentile). Decreases in perceived stress during pregnancy were also independently related to higher odds of WFA >90th percentile (OR = 1.5 (1.1, 2.0); *P* =  .01) but did not independently predict WFA *z*-scores at 6 months.

We also examined GWG as an independent predictor and potential mediator of effects of maternal prenatal behaviors on weight outcomes (Table 4). Children of normal weight mothers who were exposed to excessive GWG (*n* = 71) had higher WFA *z*-scores at birth (0.42 ± 0.75 versus 0.03 ± 0.73; *P* = 0.002) compared with children exposed to adequate GWG, but no significant effects were seen on odds of macrosomia or WFA z score >90th percentile. At 6 months, children of normal weight mothers exposed to excessive GWG had significantly (*P *= 0.03) higher odds of WFA z score >90th percentile compared with children exposed to adequate weight gain (OR = 4.0 (1.2, 13.6)), but no significant association was seen on WFA *z*-scores at 6 months (0.27 ± 0.83 versus 0.39 ± 1.1; *P* =  .28). When analyzing maternal GWG as a continuous measure, no significant effects were observed. Examining GWG as a potential mediator, the effects of maternal prenatal behaviors on offspring weight outcomes were generally attenuated (Table 4).

## 4. Discussion

This is one of the few studies to prospectively and simultaneously examine the impact of dietary, physical activity, and psychosocial parameters during pregnancy on the offspring weight status of overweight/obese and normal weight mothers. In obese mothers, we found that higher intake of sweets early in pregnancy was related to higher offspring weight status at birth and 6 months. In normal weight mothers, higher intake of soft drinks was the strongest predictor of higher offspring weight status at birth but was not a significant predictor of weight at 6 months. No prior studies have specifically examined impacts of maternal intake of sweets or soft drinks in pregnancy on offspring weight parameters. Our findings are consistent with some studies in nonpregnant adults and children showing a connection between higher intake of sweets and soft drinks and increased weight status [32], but conflicting results have been reported in association with GWG [33]. Overall, the current study extends existing work by suggesting that exposure to foods or drinks high in sugar content early in gestation may predispose offspring to higher weight status early in infancy, independent of maternal GWG. 

In obese mothers, lower protein intake early in pregnancy and increased protein intakes over the course of the pregnancy were independently related to higher offspring weight at 6 months. Studies of protein intake in pregnancy have indicated that higher maternal protein intake was positively associated with birth weight [8, 9], but there are notable exceptions [34, 35] and most studies assessed diet later in gestation. By contrast, in normal weight mothers, lower fat intake (and higher carbohydrate intake) early in pregnancy was a significant predictor of higher offspring weight at 6 months. Inverse associations between fat intake early in pregnancy and offspring weight have been reported previously [36], but not consistently [37]. Nutritional effects on the fetus and mother may vary with prepregnancy body weight, trimester of exposure during pregnancy, and overall balance of macronutrients [8, 38]. Although a recent pilot randomized trial [39] found no significant impact of a low glycemic index diet on the offspring weight of overweight/obese mothers, future, larger randomized trials and research with more frequent assessments of diet in pregnancy are needed to untangle the relationship between weight status, time in pregnancy of nutrient exposures, and offspring weight outcomes.

Surprisingly, physical activity and television viewing were not significantly related to offspring weight outcomes in normal weight or overweight/obese mothers. Low levels of physical activity and sedentary lifestyle are known contributing factors to obesity and weight gain in the general population [40]. Also, increasing physical activity during pregnancy has been related to lower GWG in some [41] but not all [42] observational studies. Few clinical trial intervention studies have specifically measured changes in physical activity during pregnancy, but available evidence suggests little to no effect on maternal GWG [16, 43]. Other research has found that a reduction in physical activity by bed rest was associated with an increase in infant birth weight [44]. Overall, findings from the current study support a stronger role for maternal intake than expenditure in predicting offspring weight outcomes, but additional studies with objective measures of physical activity (e.g., accelerometry) are clearly needed. 

Also, few maternal psychosocial predictors of offspring weight status were observed within both overweight/obese and normal weight mothers. Increases in stress were related to lower offspring weight status in normal weight mothers (adjusted analyses) and overweight/obese mothers (unadjusted analyses), which is consistent with earlier research on stress in relation to inadequate GWG [45] and long-term offspring health [46]. Overall, however, the current study's findings suggest that dietary variables may exert more of an effect than maternal psychosocial variables on offspring weight outcomes. 

Infants of overweight/obese mothers had higher WFA *z*-scores at birth than nonexposed counterparts, but the postnatal weight trajectories were different. The offspring of overweight mothers were larger at birth but, by 6-months, did not significantly differ from those of normal weight mothers. In fact the percentage of infants of normal weight mothers with WFA *z*-scores >90th percentile doubled between birth and 6-months. We found no significant prenatal predictors of change in WFA *z*-scores between birth and 6 months. Birth weight is an independent predictor of the development of obesity; however, increases in weight during the first 3–6 months of life are also influential on long-term increased risk of obesity [47, 48]. Future research is needed to determine whether differences in postnatal feeding behaviors might explain differential growth rates in offspring of normal weight and overweight/obese mothers. 

Excessive GWG was related to offspring weight but more strongly at birth than at 6 months and was not significantly related to changes in WFA *z*-scores. The association between GWG and offspring overweight may decrease as children grow older [49, 50]. Similarly, maternal prenatal behaviors may differentially impact birth and 6 months offspring weights (e.g., protein impacting 6 month but not birth weight in the current study). Interestingly, adjustment for GWG attenuated but generally did not completely remove the impact of dietary variables on offspring weight, particularly at 6 months. This suggests that dietary exposures may impact offspring weight status above and beyond the potential influence of excessive GWG. Future research with more frequent assessments of offspring weight and, potentially, analysis of growth velocities [51] might better inform how and when nutrient exposures in pregnancy impact growth.

This study is one of the first to prospectively examine impacts of prenatal diet, physical activity, and psychosocial exposures on offspring weight outcomes in a diverse sample. The study was able to adjust for several important confounds, including length of gestation and infant sex; however, the sample size was moderate, and only 69% of the offspring (mostly non-Hispanic White) had data available at 6 months. The sample was diverse but self-selected and free of diseases that might contraindicate participation in a lifestyle intervention study; thus, findings may not generalize to the population at large. A self-report, food frequency questionnaire was used, which is useful in ranking nutrient intakes of individual subjects but can be subject to bias, particularly in obese individuals [52]. The study lacked a valid measurement of infant height. Although prepregnancy weight was self-reported, our research has found that women in this study were quite accurate in recalling prepregnancy weight [16]. This study's focus was on predictors of large for gestational age at birth and 6 months, but future research is needed to examine predictors of small for gestational age, which may also predispose offspring to increased fat mass and incidence of metabolic syndrome as children and adults [53]. Also, excessive GWG was classified based on the 1990 IOM GWG guidelines; our data were collected before the newly revised 2009 IOM GWG guidelines were released. Nonetheless, analyses using the 2009 criteria confirmed the current study's findings (data not shown).

## 5. Conclusions

Findings from the current study indicate that excessive GWG and mothers' eating behaviors during pregnancy, especially intake of sweets in overweight/obese mothers, may influence offspring weight status early in infancy. More research with larger sample sizes and frequent assessments of diet is needed to inform the impact of timing of nutrient exposures on offspring weight status. Also, future adequately powered randomized clinical trials are needed to determine whether modifying maternal prenatal behaviors and GWG can prevent offspring obesity. Identifying modifiable, prenatal causes of childhood obesity will inform future interventions targeting pregnancy as a “teachable moment” for primary and secondary obesity prevention.

## Figures and Tables

**Table 1:** Characteristics of study participants and offspring.

	Overweight/obese	Normal weight	*P* value
	*N* = 132	*N* = 153	
Age, years	28.8 ± 5.1	28.2 5.5	0.30
% primiparous	66.7%	85.5	0.0001
% non-Hispanic White	62.7%	71.5	0.09
% married	68.9%	68.3	0.91
% >high school education	84.7%	86.6	0.67
% income <$25,000/year	23.7	19.0	0.27
% community clinic	28.4%	25.1	0.54
Prepregnancy BMI, kg/m^2^	30.5 ± 5.3	22.3 ± 1.8	.0001
% gain above 1990 IOM	63.8%	46.2	0.001

Infant at birth			
Weeks of gestation at birth	38.6 ± 2.5	38.7 1.9	0.14
% male	50.9%	52.4	0.83
WFA birth	0.42 ± 0.92	0.21 ± 0.76	0.02
% macrosomic (>4000 grams) at birth	17.2%	4.8	0.0001
% >90th percentile at birth	19.6%	7.1	0.001
% <10th percentile at birth	3.6%	2.2	0.53

Infant at 6 months			
WFA 6 mo	0.38 ± 1.1	0.34 ± 1.0	0.74
	*N* = 121	*N* = 128	
% >90th percentile at 6 months	17.6%	36.8	0.14
% <10th percentile at 6 months	7.6%	2.4	0.14
% mostly breastfeeding over first 6 months	28.2%	36.8	0.47

IOM: 1990 Institute of Medicine Guidelines;

WFA birth: weight for gestational age *z*-scores with Oken et al. [20] reference data;

WFA 6 mo: weight for age, sex-specific *z*-scores with 2000 Centers for Disease Control reference data.

**Table 2:** Significant, unadjusted correlations between prenatal variables and WFA *z*-scores in offspring of overweight/obese mothers.

Maternal variables	Offspring WFA z	Offspring WFA *z*-scores
	scores at birth	at 6 months
Early in pregnancy		
% kcal from protein	ns	−0.31, *P* = 0.001
% kcal sweets	0.19, *P* = 0.01	0.36, *P* = 0.0001
Dietary restraint	ns	−0.22, *P* = 0.02
Disinhibition	0.17, *P* = 0.03	ns
Perceived stress	0.18, *P* = 0.02	0.18, *P* = 0.05
Fast food consumption	ns	0.21, *P* = 0.03
During pregnancy		
Increases in dietary restraint	−0.17, *P* = 0.004	ns
Increases in stress	ns	−0.21, *P* = 0.03
Increases in % kcal from carbohydrates	0.18, *P* = 0.03	ns
Increases in % kcal from fat	−0.22, *P* = .007	ns
Increases in % kcal from protein	ns	0.23, *P* = .01

Ns: non-significant, *P ≥*.05;

WFA: weight for age.

**Table 3:** Final regression models predicting weight for age *z*-scores in offspring of overweight/obese mothers at birth (*N* = 132) and 6 months (*N* = 121).

Final model predicting weight for age *z*-scores at birth					
	B	CI for B	Beta	*P* value*	*P* value GWG**
Prepregnancy BMI	0.005	−0.02, 0.03	0.03	0.73	0.03
Multiparity	0.20	−0.11, 0.50	0.10	0.21	0.13
% kcal from sweets early in preg	0.02	0.003, 0.04	0.19	0.004	0.06
Perceived stress early in preg	0.04	−0.02, 0.09	010	0.18	0.16

Final model predicting weight for age *z*-scores at 6 months					

Prepregnancy BMI	0.009	−0.03, 0.05	0.044	0.639	0.42
Non-Hispanic White (ref: others)	0.12	−0.49, 0.72	0.047	0.706	0.74
% kcal from sweets early in pregnancy Increases in % kcal from protein in preg	0.04	0.02, 0.06	0.296	0.002	0.006
Restraint early in pregnancy	−0.04	−0.09, 0.01	−0.144	0.140	0.12
Increases in perceived stress	0.06	−0.02, 0.13	0.146	0.124	0.19

GWG: gestational weight gain; preg: pregnancy;

BMI: body mass index.

*Adjusted for infant sex, treatment group, recruitment site, weeks of gestation at delivery; 6-month analyses also adjusted for breastfeeding.

**Adjusted for infant sex, treatment group, recruitment site, weeks of gestation at delivery, and total maternal gestational weight gain; 6-month analyses also adjusted for breastfeeding.

Overall omnibus test at birth: *F *= 3.0; *P* =  0.004 (with GWG: *F* = 5.6; *P*  =  0.0001); at 6 months: *F* = 4.1; *P*  =  .0001 (with GWG: *F* = 3.9; *P*  =  .0001).

**Table 4:** Final regression models predicting weight for age *z*-scores in offspring of normal weight mothers at birth (*N* = 153) and 6 months (*N* = 128).

Final model predicting weight for age *z*-scores at birth					
	B	CI for B	Beta	*P* value*	*P* value GWG**
Prepregnancy BMI	0.02	−0.04, 0.09	0.06	0.43	0.69
Multiparity	0.33	0.02, 0.63	0.15	0.004	0.04
Non-Hispanic White (others: ref)	0.35	0.68, 0.009	0.21	0.05	0.07
Daily calories from soft drinks	0.002	0.0001, 0.004	0.16	0.04	0.10

Final model predicting weight for age *z*-scores at 6 months					

Prepregnancy BMI	0.04	−0.05, 0.13	0.08	0.34	0.31
Multiparity	0.62	0.01, 1.2	0.18	0.05	0.04
% kcal from fat early in preg	−0.06	−0.09, −0.03	−0.35	0.0001	0.001
% kcal from sweets early in preg	−0.02	−0.04, 0.003	−0.15	0.10	0.14

GWG: gestational weight gain; preg: pregnancy; BMI: body mass index.

*Adjusted for infant sex, treatment group, recruitment site, weeks of gestation at delivery; 6-month analyses also adjusted for breastfeeding.

**Adjusted for infant sex, treatment group, recruitment site, weeks of gestation at delivery, and total maternal gestational weight gain; 6-month analyses also adjusted for breastfeeding.

Overall omnibus test at birth: *F* = 3.4; *P* = 0.001 (with GWG: *F* = 3.6; *P *= 0.0001); at 6 months: *F* = 3.6; *P*  =  .001 (with GWG: *F* = 3.3; *P *= 0.001).
